# Cryopreservation of clonal and polyclonal populations of *Chlamydomonas reinhardtii*

**DOI:** 10.1093/biomethods/bpab011

**Published:** 2021-06-21

**Authors:** Jacob Boswell, Charles Ross Lindsey, Emily Cook, Frank Rosenzweig, Matthew Herron

**Affiliations:** Division of Biological Sciences, University of Montana, Missoula, MT 59812, USA; School of Biological Sciences, Georgia Institute of Technology, Atlanta, GA 30336, USA; School of Biological Sciences, Georgia Institute of Technology, Atlanta, GA 30336, USA; School of Biological Sciences, Georgia Institute of Technology, Atlanta, GA 30336, USA; Division of Biological Sciences, University of Montana, Missoula, MT 59812, USA; School of Biological Sciences, Georgia Institute of Technology, Atlanta, GA 30336, USA; Division of Biological Sciences, University of Montana, Missoula, MT 59812, USA; School of Biological Sciences, Georgia Institute of Technology, Atlanta, GA 30336, USA

**Keywords:** *Chlamydomonas reinhardtii*, cryopreservation, GeneArt™, −80°C, clonal, polyclonal

## Abstract

Long-term preservation of laboratory strains of *Chlamydomonas reinhardtii* has historically involved either liquid nitrogen cryopreservation, which is expensive and labor intensive, or storage on agar plates, which requires frequent transfer to new plates, and which may leave samples susceptible to contamination as well as genetic drift and/or selection. The emergence of *C. reinhardtii* as a model organism for genetic analysis and experimental evolution has produced an increasing demand for an efficient method to cryopreserve *C. reinhardtii* populations. The GeneArt™ Cryopreservation Kit for Algae provides the first method for algal storage at −80°C; however, little is known about how this method affects recovery of different clones, much less polyclonal populations. Here, we compare postfreeze viability of clonal and genetically mixed samples frozen at −80°C using GeneArt™ or cryopreserved using liquid nitrogen. We find that the GeneArt™ protocol yields similar percent recoveries for some but not all clonal cultures, when compared to archiving via liquid N2. We also find that relative frequency of different strains recovered from genetically mixed populations can be significantly altered by cryopreservation. Thus, while cryopreservation using GeneArt™ is an effective means for archiving certain clonal populations, it is not universally so. Strain-specific differences in freeze–thaw tolerance complicate the storage of different clones, and may also bias the recovery of different genotypes from polyclonal populations.

## Introduction

*Chlamydomonas reinhardtii* is a single-celled, chlorophyte alga with a well-annotated genome, reliable methods for genetic manipulation, an extensive library of mutants, and a rich history of experimental investigation [1]. As a model organism, *C. reinhardtii* has been used to shed light on biological processes ranging from photosynthesis and cell motility to taxis and sexual reproduction [2–6]. *Chlamydomonasreinhardtii* has also been the subject of research into biofuel production [7] and into the genetics of adaptation during laboratory experimental evolution [7–9]. Each of these different lines of inquiry requires the use of archived samples that consist of either single strains or mixtures of strains produced by induced or spontaneous mutation. A reliable method for archiving either isogenic or heterogenic cell cultures in a state where they are genetically stable and can be easily distributed would accelerate research along multiple fronts.

Before the advent of the first reliable method of cryopreservation, *C. reinhardtii* clones were stored either in liquid batch culture or as colonies on agar plates at low temperature [1, 10]. These procedures allow cell lines to be stored for up to 6 months but require cells to be transferred to fresh liquid or solid medium, leaving populations susceptible to genetic drift and selection [11] and references therein, as well as to bacterial contamination [10]. The development of a liquid nitrogen (LN_2_) cryopreservation protocol [12], and subsequent modifications of that protocol utilizing dimethyl sulfoxide and methanol reagents [13–16] represent an improvement over these older methods, but at a significant cost in labor and materials. A simple, reliable method of cryopreservation at –80°C, such as those used routinely with other microbial models, has long been desired. The GeneArt™ Cryopreservation Kit for Algae (Catalog # A24228, Thermo Fisher Scientific) provides the first method for cryopreserving *C. reinhardtii* at −80°C that allows archived algae to be stored in Ultra-Low Temperature freezers. However, little is known about how the GeneArt™ protocol affects relative recovery of different laboratory strains, much less how it may affect recovery of genetically heterogeneous populations of *C. reinhardtii* produced by mutagenesis or by laboratory evolution. Recent work on cryopreservation of mixed populations of laboratory-evolved Bakers’ yeast [17] and Escherichia *coli* [18] suggests that differential survival can alter genotypic frequencies, with consequences for the evolutionary trajectory of populations revived from storage at −80°C. To fill these knowledge gaps, we assessed a previously unreported aspect of the kit’s efficacy: the survival rate of an isogenic (i.e. clonal) sample during freeze–thaw in comparison with the survival rates of different strains present in a heterogenic (polyclonal) sample. We then compared over a 15-day period the relative recovery of *C. reinhardtii* cells with clonal and polyclonal cultures cryopreserved using either the GeneArt™ protocol or the LN_2_ protocol of Sayre [19].

## Materials and methods

### Strains and culture conditions

Strains were obtained from the Chlamydomonas Culture Collection, University of Minnesota (Chlamydomonas Resource Center, RRID: SCR_014960), see Table 1. Reference wild-type strain CC-1690 (Sager 21 gr), mt+ [20, 21] was used for the isogenic (i.e. monoclonal) cultures, and five strains with differing antibiotic resistances were used for pooled polyclonal cultures. Strain CC-87, nr-u-2-1 mt-, is resistant to kanamycin (Kan) due to an A→G change at nucleotide 1340 of the chloroplast 16S rRNA gene as described in Harris *et al.* [22]. Strain CC-119, sr-u-2-60 mt-, is resistant to streptomycin (Str) due to an A → C change at base 474 of 16S rRNA gene as described in Harris *et al.* [22]. Strain CC-504, ery1b mt+, with resistance to antibiotic erythromycin (Ery), is also suspected to harbor a pf (paralyzed flagella) mutation as very few swimming cells are observed in culture [23, 24]. CC-2355, CAP9 mt+, is resistant to chloramphenicol (Chl) [25] due to a change in the RIB1 region of the 23S chloroplast ribosomal RNA, and lastly strain CC-3673, ani1 mt-, was generated at Duke University in 1998 from a cross of CC-399 × CC-124 and selected for resistance to anisomycin (Ani).

**Table 1:** Phenotypes of *Chlamydomonas* strains used in this study

Strain	Antibiotic resistance	Genetic cause of antibiotic resistance	Mating type	Citation(s)
CC-87	Kan	A to G change at base 1340 of chloroplast 16S rRNA gene	Mt-	[22]
CC-119	Str	A to C change at base 474 of 16S rRNA gene	Mt-	[22]
CC-504	Ery	Altered protein in large subunit of chloroplast ribosome	Mt+	[23, 24]
CC-1690	None determined		Mt+	[20, 21]
CC-2355	Chl	Change in RIB1 region of 23S chloroplast rRNA	Mt+	[25]
CC-3673	Ani		Mt-	Chlamydomonas Genetics Center, Duke University, 1998

Unless otherwise noted, cells were cultured in Tris Acetate Phosphate (TAP) medium (Tris Acetate Phosphate, Thermo Fisher Scientific, Catalog # A1379801) and incubated at 23°C with a 14-h:10-h light:dark cycle on a rotary shaker at 110 rpm. Cell culture densities were measured by direct counts on a hemocytometer observed using a Zeiss Axiovert.A1 microscope ×10 objective

Antibiotics were sourced as follows: Anisomycin (Catalog #A9789), Erythromycin (Catalog # E5389), and Str (Catalog # S9137) from Sigma Aldrich; Kanamycin sulfate from Fisher Scientific (Catalog # BP906-5) and Chloramphenicol from VWR (Catalog # 97061-244).

### Clonal survival assay using GeneArt™

An isogenic culture of *C. reinhardtii* strain CC-1690 was inoculated into 150 ml of TAP medium and grown for 3 days. As outlined in the GeneArt™ Cryopreservation Kit for Algae manufacturer’s protocol, samples of this culture were diluted into triplicate vials of precondition medium (45 ml TAP:1 ml Reagent A) to a final cell density of 1.3 × 10^5^ cells ml^−1^. The preconditioning medium was incubated for an additional 3 days under the same environmental conditions as above, after which cell density was measured in triplicate by hemocytometer to be within 2.7–2.8 × 10^6^ cells ml^−1^ for each of the replicates. Each sample was separated into two vials: one to be cryopreserved and one to serve as a nonfrozen control. All control and experimental samples were centrifuged at 1452*g*for 5 min, then the supernatant was removed and the resulting cell pellets were suspended in either Reagent B (experimental samples) or TAP media (control samples) to a final concentration of 2.5 × 10^7^ cells ml^−1^.A small volume of each control sample was removed and serially diluted to 2000 cells ml^−1^, then 50 μl of this dilution was spread plated onto TAP agar in triplicate, such that approximately 100 colonies might be observed upon growth. After incubating experimental samples at room temperature for 40 min, 240 μl aliquots were transferred into three cryovials per sample, and placed directly into −80°C storage, inside an empty Mr Frosty freezing container (Catalog #15-350-50, Thermo Fisher). After 3 days, the cryovials were removed from cold storage and thawed for 1 min in a 35°C water bath. Each sample was diluted to a concentration of 2000 cells ml^−1^, then 50 μl of these dilutions were plated onto TAP agar in triplicate for colony counting.

### Polyclonal survival assay using GeneArt™

Five uniquely antibiotic-resistant strains of *C. reinhardtii* (CC-87, CC-119, CC-504, CC-2355, CC-3673; Table 1) were inoculated into 150 ml TAP in separate 250 ml flasks and incubated to log-phase growth under the culture conditions described above. As outlined by the GeneArt™ Cryopreservation Kit for Algae, each was inoculated into preconditioning medium (45 ml TAP: 1 ml Reagent A) to a final concentration of 1.3 × 10^5^ cells ml^−1^. After 4 days of growth, the cell density of each strain was measured by hemocytometer, and equal cell numbers from each strain were mixed into a final volume of 45 ml. A control sample was removed from this culture and plated directly onto TAP agar at several dilutions for strain identification. The remaining volume was centrifuged at 1452*g* for 5 min, the supernatant was discarded, and the cell pellets were resuspended in Reagent B to a final concentration of 2.5 × 10^7^ cells ml^−1^. After incubation at room temperature for 40 min, 240 μl aliquots of the sample were transferred to cryovials and placed into −80°C storage inside an empty Mr Frosty™ freezing container. After at least 4 days at −80°C, each cryovial was removed from storage and thawed for 1 min in a 35°C water bath. The thawed sample was then plated onto agar at various dilutions for strain identification.

**Figure 1:**
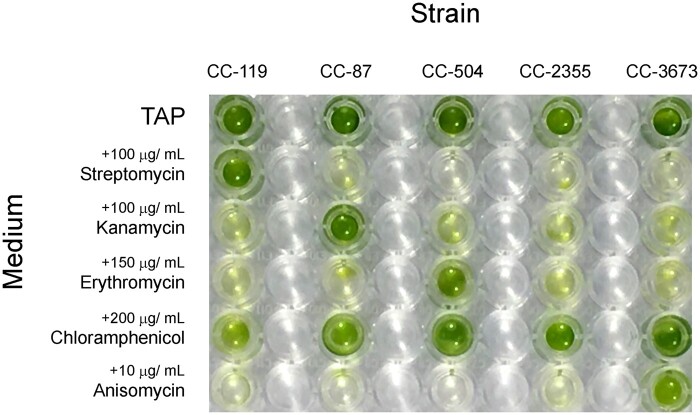
Different *C. reinhardtii* strains exhibit characteristic patterns of antibiotic resistance and sensitivity. An individual strain’s characteristic pattern enables it to be identified in mixed culture and its frequency determined relative to other strains present. The Chl resistance of strain CC-2355 is best ascertained during heterotrophic (dark) growth, but in order to screen polyclonal cultures in parallel a light–dark cycle was utilized. Although this slightly diminished the stringency of the selection, the growth differences were clearly distinguishable by eye.

After 5 days of growth on TAP agar at 23°C on a 14:10 light cycle, 205 colonies were randomly selected from each set of both control and experimental plates, and every colony was inoculated into a 5 ml test tube of fresh TAP. As each strain exhibits a unique antibiotic-resistance profile, strain identification could easily be performed by testing viability against a panel of antibiotics (Fig. 1). To do this, each row of a 96-well plate was filled with a 200 µl volume of either TAP medium, TAP + 100 µg ml^−1^ Str, TAP + 100 µg ml^−1^ Kan, TAP + 150 µg ml^−1^ Ery, TAP + 200 µg ml^−1^ Chl, or TAP + 10 µg ml^−1^ Ani. To every second column, we inoculated with 50 μl of dense (∼3 × 10^6^ cells ml^−1^) culture, the first column serving as a sterile medium control. A strain’s pattern of growth in relation to its known antibiotic susceptibilities revealed its identity. In rare instances where the pattern of growth was ambiguous, the sample was discarded from the analysis.

### Method for reduced volume reactions

In an effort to conserve costly reagents, reduced volume reactions were used throughout the course of our experiments. According to the manufacturer’s protocol, a single kit is designed for the preservation of five strains, each with 20 separate aliquots. By reducing the volume of the preconditioning medium from 46 ml (45 ml TAP: 1 ml Reagent A) to 15.333 ml (15 ml TAP: 0.333 ml Reagent A), the utility of a single kit could be expanded from 5 to 15 strains. All other instructions in the manufacturer’s protocol remain unchanged. Reduced volume reactions have been successfully used to archive model strains CC-87, CC-119, CC-504, CC-1690, CC-2290, CC-2355, and CC-3673, as well as strains experimentally evolved in our laboratory: B2-01, B2-03, B2-04, B2-10, B2-11, B5-05, B5-06, K1-01, and K1-06 [8, 26] without failure.

### Long-term survivorship following either GeneArt™ or liquid nitrogen cryopreservation

#### Monoclonal survival assays

*GeneArt*^TM^*Monoclonal Survival Assay:* Isogenic cultures of *C. reinhardtii* strains CC-1690, CC-87, and CC-119 were inoculated into 150 ml of TAP medium and grown for 3 days. According to the GeneArt^TM^ Cryopreservation Kit for Algae manufacturer’s protocol, samples from each culture were diluted into flasks of preconditioning medium (15 ml TAP: 0.333 ml Reagent A) to a final cell density of 1.3 × 10^5^ cells ml^−1^. Cells in preconditioning medium were incubated for additional 4 days, then cell density of each culture was measured by hemocytometer. Cell densities for CC-1690, CC-87, and CC-119 were measured to be: 5 × 10^6^ cells ml^−1^, 1.06 × 10^7^ cells ml^−1^, and 6.8 × 10^6^ cells ml^−1^, respectively. An appropriate amount of each culture was pipetted into a 15 ml Falcon tube and pelleted via centrifuge at 1452*g* for 5 min in order to achieve a final cell density in Reagent B of 3.5 × 10^7^ cells ml^−1^. After incubating each sample at room temperature for 40 min, 240 μl aliquots were transferred into four cryovials per sample and placed directly into −80°C storage, inside an empty Mr Frosty freezing container (Catalog #15-350-50, Thermo Fisher). Cryovials for each sample were thawed at four time points: 1, 5, 10, and 15 days postfreezing. Day 15 was selected as a final time point as percent viability had been previously reported as qualitatively equivalent between samples stored for 2 weeks to those stored for 2 years [13]. Once thawed, each sample was diluted to 1 ml and 1:10, 1:100, and 1:1000 serial dilutions were performed and 100 μl were plated onto TAP agar to determine viable cell counts. Equivalent serial dilution and plating of control samples were performed prior to freezing in order to determine viable cell counts.

*LN2Monoclonalsurvivalassay:* Isogenic cultures of *C. reinhardtii* strains CC-1690, CC-87, and CC-119 were inoculated into 150 ml of TAP medium and allowed to grow for 3 days to cell densities of 4.15 × 10^6^ cells ml^−1^, 2.45 × 10^6^ cells ml^−1^, and 3.23 × 10^6^ cells ml^−1^, respectively. A 6% methanol TAP solution was prepared. Four cryovials per sample were prepared by mixing cells from each culture 1:1 with the 6% methanol TAP solution (250 µl each, 3% methanol final concentration). Cryovials were placed in a Mr Frosty freezing container with 250 ml isopropanol and transferred to a −80°C freezer for 70 min. Cryovials were then transferred to a LN_2_ storage dewar. Cryovials for each sample were thawed at four time points: 1, 5, 10, and 15 days postfreezing. Cryovials were rapidly thawed in a 35°C water bath. Once thawed, the contents of each cryovial were decanted into a 15 ml falcon tube with 5 ml of room temperature TAP media. Cells were pelleted by centrifugation at 1452*g* for 5 min. The supernatant was removed, and cells were resuspended in 1 ml of TAP media and allowed to sit under hood lights for 6 h. After allowing the cell suspension to sit under hood lights for 6 h, 1:10, 1:100, and 1:1000 serial dilutions were performed, and 100 μl were plated onto TAP agar to determine viable cell counts. Equivalent serial dilution and plating of control samples were performed prior to freezing in order to determine viable cell counts.

#### Polyclonal survival assay

*GeneArt*^TM^*polyclonalsurvivalassay:* Isogenic cultures of *C. reinhardtii* strains CC-87 and CC-119 were inoculated into 150 ml of TAP medium and grown for 3 days. Following the GeneArt^TM^ Cryopreservation Kit for Algae manufacturer’s protocol, samples from each culture were diluted into flasks of preconditioning medium (15 ml TAP: 0.333 ml Reagent A) to a final cell density of 1.3 × 10^5^ cells ml^−1^. Cells in preconditioning medium were incubated for additional 4 days, then cell density of each culture was measured by hemocytometer. Cell densities for CC-87 and CC-119 were measured to be: 1.06 × 10^7^ cells ml^−1^ and 6.8 × 10^6^ cells ml^−1^, respectively. An appropriate amount of each culture was pipetted into a 15 ml Falcon tube and pelleted via centrifugation tiu at 1452*g* for 5 min in order to achieve a final cell density in Reagent B of 3.5 × 10^7^ cells ml^−1^. Polyclonal culture consisting of CC-87 and CC-119 was mixed prior to freezing the monoclonal cultures. Approximately equal numbers of cells from each strain were mixed according to hemocytometer counts. After incubating each sample at room temperature for 40 min, 240 μl aliquots were transferred into four cryovials, and placed directly into −80°C storage, inside an empty Mr Frosty freezing container (Catalog #15-350-50, Thermo Fisher). Cryovials for the polyclonal culture were thawed at four time points: 1, 5, 10, and 15 days postfreezing. Once thawed, each sample was diluted to 1 ml and 1:10, 1:100, and 1:1000 serial dilutions were performed and 100 μl were plated onto TAP agar to determine viable cell counts. Equivalent serial dilution and plating of control samples were performed prior to freezing in order to determine viable cell counts.

*LN_2_polyclonalsurvivalassay:* Polyclonal culture consisting of CC-87 and CC-119 was mixed prior to freezing the monoclonal cultures. Approximately equal numbers of cells from each strain were mixed according to hemocytometer counts. A 6% methanol TAP solution was prepared. Four cryovials were prepared by mixing cells from the polyclonal culture 1:1 with the 6% methanol TAP solution (250 µl each, 3% methanol final concentration). Cryovials were placed in a Mr Frosty freezing container with 250 ml isopropanol and transferred to a −80°C freezer for 70 min. Cryovials were then transferred to LN_2_ storage dewar. Cryovials were thawed at four time points: 1, 3, 5, and 15 days postfreezing. Cryovials were rapidly thawed in a 35°C water bath. Once thawed, the contents of each cryovial were decanted into a 15 ml falcon tube with 5 ml of room temperature TAP medium. Cells were pelleted by centrifugation at 1452*g* for 5 min. The supernatant was removed, and cells were resuspended in 1 ml of TAP media and allowed to sit under hood lights for 6 h. After allowing the cell suspension to sit under hood lights for 6 h, 1:10, 1:100, and 1:1000 serial dilutions were performed and 100 μl were plated onto TAP agar to determine viable cell counts. Equivalent serial dilution and plating of control samples were performed prior to freezing in order to determine viable cell counts.

To determine strain frequency for polyclonal cultures cryopreserved under both methods, 96 colonies were randomly picked from a serial dilution plate for each time point. Each set of colonies was grown in a 96-well plate with 200 µl TAP in each well. After 3 days of growth in the 96-well TAP plate, 5 µl of culture was pipetted into two other 96-well plates that contained either TAP + 100 µg/ml Kan or TAP + 100 µgml^-1^ Str. Four to 5 days were allowed for culture growth in 96-well plates containing antibiotic, and wells with growth were counted for either Kan- or Str-resistant strains.

## Results

### Effect of cryopreservation on the viability of a clonal sample

Preservation of *C. reinhardtii* in LN_2_ has been previously described by differing methodologies [12–16] reporting quite varied postthaw cell viabilities, ranging from 0.02 to 75% recovery. The GeneArt™ protocol boasts an impressive 100% postthaw viability for *C. reinhardtii* and *Chlorella.* We tested these claims in *C. reinhardtii* by directly comparing growth of algal strain CC-1690 on TAP agar from paired pre- and postfreeze samples. Specifically, we tested the hypothesis that the number of colony-forming units (CFUs) observed on agar was different between the prefreeze and postthaw samples using a paired two-sample *t*-test. In addition to this test, a 95% confidence interval was constructed for the difference in means between the two treatments, from which a survival rate could be estimated.

**Figure 2:**
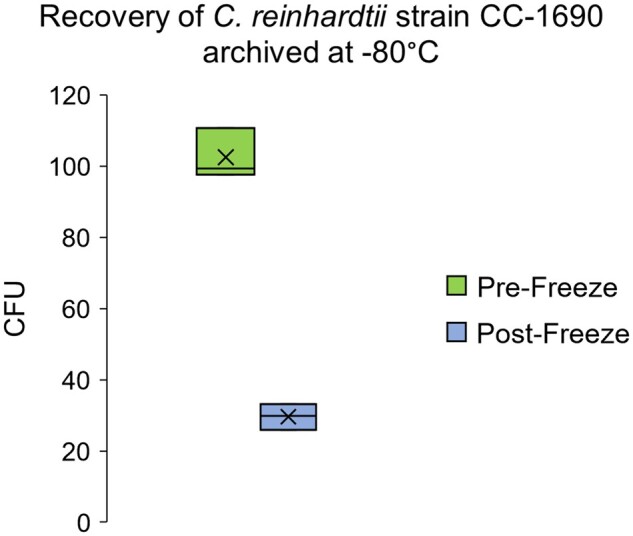
Recovery of *C. reinhardtii* strain CC-1690 cryopreserved at −80°C using the GeneArt™ protocol. Distribution of CFU (colony forming unit) counts observed from triplicate paired pre- and postfreeze samples. Box height indicates the range of values observed among replicates, pre- and postfreeze. Horizontal lines across bars represent median values; *x*’s within bars represent means. Significantly fewer colonies were recovered from samples of equal density that had been cryopreserved relative to those that had not (paired *t*-test, *P *<* *9.5 × 10^−4^).

The results of a paired *t*-test showed a significant decrease in the number of CFU observed on agar after GeneArt™ cryopreservation (*P* <9.5 × 10^−4^). A 95% confidence interval for the difference between the means of the two treatment groups was calculated at 72.9 ± 12.0 CFU. On an average ∼30% of CC-1690 cells survived cryopreservation at −80°C (Fig. 2).

### Effect of cryopreservation on polyclonal samples

We also tested the hypothesis that the relative frequencies of different strains in a polyclonal sample were altered during cryopreservation by using repeated *G*-tests of goodness-of-fit. These tests compared the observed distributions of strains in the post freeze sample groups with the observed distributions in the prefreeze sample groups, and generated statistics for hypothesis testing. First, each paired group of pre- and postfreeze samples was tested individually for significant differences, then samples were pooled together and tested for significant differences collectively.

**Figure 3:**
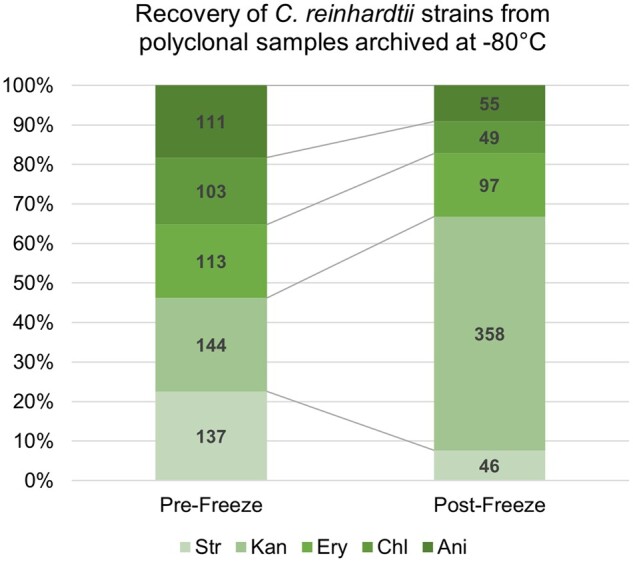
Recovery of different *C. reinhardtii* strains from polyclonal samples cryopreserved at −80°C using the GeneArt™ protocol. Strain frequencies were pooled from triplicate paired pre- and postfreeze samples. Absolute counts of CFU are shown within bars.

The results of repeated *G*-tests of goodness-of-fit indicated that for each set of paired samples, and for the pooled samples as a whole, the strain frequencies in a polyclonal sample were significantly altered by cryopreservation. Furthermore, the direction and magnitude of the change in strain frequencies were largely repeatable (Fig. 3 and Table 2). For each set of observations, the null hypothesis that the post freeze strain frequencies are equal to the prefreeze strain frequencies was rejected. In each case, a significantly higher proportion of Kan-resistant (CC-119) isolates was recovered postfreeze. When strains are pooled together, the significance of this result increases dramatically (Table 2).

**Table 2:** Pre- and postfreeze recovery of different *C. reinhardtii* strains from polyclonal cultures

Replicate	Treatment	Str	Kan	Ery	Chl	Ani	df	*G*	*P*-value
1	Postfreeze	15	113	27	21	27	4	67.2	<0.00001
	Prefreeze	33	59	47	33	32			
2	Postfreeze	13	113	41	20	11	4	240.8	<0.00001
	Prefreeze	50	28	36	41	48			
3	Postfreeze	18	132	29	8	17	4	132.5	<0.00001
	Prefreeze	54	57	30	29	31			
Pooled	Postfreeze	46	358	97	49	55	4	378.11	<0.00001
	Prefreeze	137	144	113	103	111			

Five different strains can be discriminated on the basis of their antibiotic resistance and sensitivities (Table 1 and Fig. 1). We performed statistical analysis of approximately 200 randomly selected colonies from both pre- and postfreeze plating (as described in ‘Polyclonal survival assay’ section). Repeated *G*- tests of goodness-of-fit indicate that cryopreservation using the GeneArt™ protocol biases recovery of different genotypes.

### Long-term survivorship of *C. reinhardtii* following either GeneArt^TM^ or LN_2_ cryopreservation

**Figure 4:**
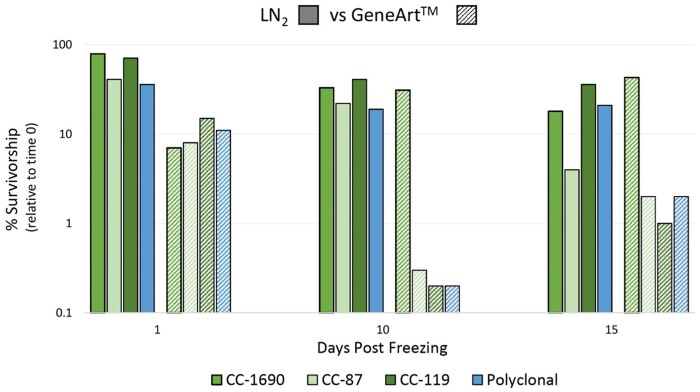
Percent recovery of monoclonal and polyclonal cultures for LN2 and GeneArt™ cryopreservation methods. Percent recovery for monoclonal and polyclonal cultures cryopreserved using the LN2 method (solid columns) and GeneArt™ kit (hatched columns). The time point for 5 days postfreezing is not shown due to human error during the thawing process of the LN2 samples. The polyclonal sample consists of strains CC-87 and CC-119.

Strain collections typically consist of individual clones, whereas mutagenesis and evolution experiments typically produce mixtures of clones. Cryopreservation of either sample type is aimed at creating a long-term resource. The experiments described in sections ‘Effect of cryopreservation on the viability of a clonal sample’ and ‘Effect of cryopreservation on polyclonal samples’ were conducted over 72 h using GeneArt^TM^ only. To assess the relative efficacy of each method for long-term storage, we cryopreserved single clones and mixtures of these clones using either the GeneArt^TM^ protocol or the LN_2_ protocol of Sayre [19], then assayed cells’ survivorship over a 15-day interval (Fig. 4).

Overall, we found that the LN_2_ protocol (Fig. 4, solid columns) allowed for higher percent recovery of all monoclonal cultures relative to the same strains prepared using GeneArt^TM^ (Fig. 4, hatched columns). CC-1690 and CC-119 responded better to cryopreservation under LN_2_ than did strain CC-87 (Fig. 4). After 15 days, CC-119, CC-1690, and CC-87 exhibited survivorships of 36, 18, and 4%, respectively, whereas survivorship of polyclonal cultures was 21%. With the exception of CC-1690, long-term survivorship of *C. reinhardtii* strains cryopreserved using GeneArt^TM^ was poor (Fig. 4, hatched columns). In contrast, survivorship of CC-1690 using GeneArt^TM^ was within range values reported in both Figs 2 and 4.

**Figure 5:**
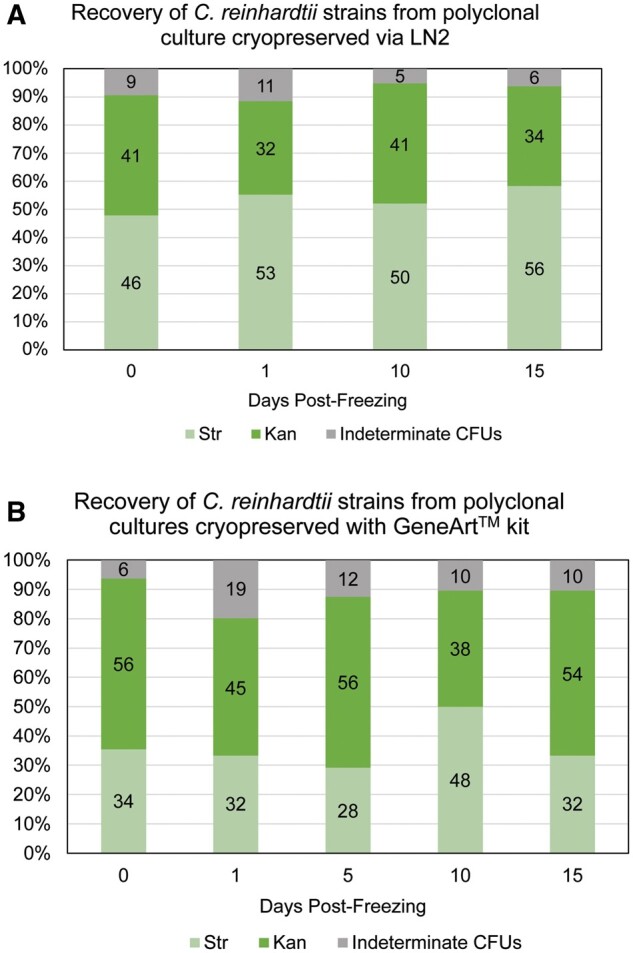
Recovery of *C. reinhardtii* strains from polyclonal cultures cryopreserved using the LN2 and GeneArt™ protocols. (A) Strain recoveries from polyclonal samples cryopreserved under the LN2 protocol. (B) Strain recoveries from polyclonal samples cryopreserved using the GeneArt™ kit. Absolute counts of CFU are shown within the bars. Kan-resistant strain, which is CC-87. Str-resistant strain, which is CC-119.

We further tested whether long-term cold storage biased recovery of genetically different *C. reinhardtii* strains. For this experiment, we chose the Kan-resistant strain, CC-87, and the Str-resistant strain, CC-119. These strains exhibited pronounced differences in survivorship as clones using the LN_2_ protocol, though they were indistinguishable using GeneArt™. Interestingly, over a 15-day interval the relative proportion of genotypes recovered did not change dramatically from the proportions scored prior to freezing using either LN_2_ or GeneArt™ (Fig. 5A and B). This result stands in contrast with results presented in Fig. 3 where polyclonal cultures cryopreserved using GeneArt^TM^ were stored for ∼72 h at −80°C. We speculate that this discrepancy may be attributable to the use of only two rather five input strains.

## Discussion

Our experiments indicate that the GeneArt™ Cryopreservation Kit for Algae can provide a short-term storage alternative to agar plates or to LN_2_ cryopreservation for clonal samples; for some strains (e.g. CC-1690), the procedure may also be suitable for long-term storage. CC-1690, a member of the Sager lineage [27], is a widely adopted laboratory strain that was used to create EST libraries for cDNA sequencing in the Chlamydomonas Genome project [21]. Our GeneArt™ data for this strain contrast with a recent study that found postfreeze viability of *C. reinhardtii* in GeneArt™ reagents was dramatically lower than that obtained by LN_2_ preservation, as measured by a postthaw growth curve in liquid TAP [28]. It is noteworthy that the authors of [28] measured the growth rate of a population after recovery from cryopreservation, whereas we measured absolute numbers of surviving cells after recovery. Our finding that a relatively high number of CC-1690 cells survive despite initially slower growth suggests a transient period of reduced reproductive capacity in surviving cells after they are revived. The discordance between these results may also be attributed to the use of different strains or different culture conditions. Overall, we find that the survivorship of CC-1690 at −80°C (ca. 25%) is in the range of values previously reported for LN_2_ (5–40%) [13, 15, 29]. In contrast, we found that two other laboratory strains, CC-119 and CC-87, responded poorly to GeneArt™ cryopreservation; for these strains, our data agree with the assessment given by Scarbrough and Wirschell [28]. Thus, prior to considering use of this protocol for any other strain than CC-1690, researchers are urged to empirically estimate survivorship.

A mixture of genotypes (a polyclonal sample) can be expected to arise in a variety of research contexts including samples obtained from environmental sources, from laboratory experimental evolution, or from samples that have been mutagenized. Our data show that different genotypes can exhibit different levels of survivorship following cryopreservation, regardless of which protocol is employed. Thus, the relative abundance of different genotypes in genetically heterogeneous samples may not be reliably conserved in frozen stocks. Differences in freeze–thaw tolerance may be explained by among-strain genetic differences governing this trait; indeed, such differences have been shown among strains of other microbes [30–32]. In the bacterium *E.coli*, freeze–thaw tolerant strains have been generated by IS150 (transposon) insertions into the *cls* gene, which codes for an essential phospholipid utilized in the cell membrane, as well as by IS150 insertions into the *uspA-uspB* intergenic region, which is flanked by two universal stress–response proteins. Further experimentation revealed that the mutations in *cls* conferred increased membrane fluidity in the culture conditions of the experiment, permitting tolerance to temperature fluctuations [33]. *Chlamydomonasreinhardtii* strains used in the experiments reported here were intentionally chosen to be distantly related so that we could test for the effect of genetic heterogeneity. The differences we report in strains’ tolerance to freezing may be related to differences in their capacities to form plasmalemma structures associated with freeze tolerance [34], or to differences in stress–response proteins, such asAsp-Glu-Ala-Asp (DEAD)-box RNA helicase proteins [35] and Hsp70, variants of which appear to underlie the psychrophilic habit of Antarctic sea-ice *Chlamydomonas* sp. ICE-L [35, 36].

For researchers using monoclonal strains, low postfreeze viability may not be an important issue as long as some cells survive. However, our results serve as a cautionary tale to investigators working with mixed *C. reinhardtii* populations who may need to recover genotypes from cryopreserved samples at their original frequencies. In addition, our data further illustrate how genotypic variation can drive major differences in algal responses to stress in the form of freeze–thaw tolerance.
